# Gap modification of atomically thin boron nitride by phonon mediated interactions

**DOI:** 10.1186/1556-276X-7-303

**Published:** 2012-06-14

**Authors:** James P Hague

**Affiliations:** 1 Department of Physical Sciences, The Open University, Walton Hall, Milton Keynes, MK7 6AA, UK

**Keywords:** Boron nitride, Electron-phonon interactions, Semiconductors, Two-dimensional materials, Graphene

## Abstract

A theory is presented for the modification of bandgaps in atomically thin boron nitride (BN) by attractive interactions mediated through phonons in a polarizable substrate, or in the BN plane. Gap equations are solved, and gap enhancements are found to range up to 70% for dimensionless electron-phonon coupling *λ *=1, indicating that a proportion of the measured BN bandgap may have a phonon origin.

## Background

The need for bandgaps in graphene on electronvolt scales has led to a number of proposals, such as the use of bilayer graphene [1], creation of nanoribbons [2], and manipulation through substrates [3,4]. Recently, it has become possible to manipulate atomically thin layers of boron nitride (BN) and other materials with structure similar to graphene [5]. This may lead to a complimentary method of manipulating bandgaps to make digital transistors.

In low dimensional materials, strong effective electron-electron interactions can be induced via an interaction between electrons confined to a plane and phonons in a polarizable neighboring layer [6]. The theory has shown that similar interactions account for the transport properties of graphene on polarizable substrates [7] and that sandwiching graphene between polarisable superstrates and gap opening substrates can cause gap enhancement [8]. This paper examines similar gap changes in atomically thin BN due to interactions mediated through substrates.

## Methods

Atomically thick hexagonal BN (h-BN) has similar chemistry to graphene: bonding occurs through *sp*_2_ hybridization, and electrons with energies close to the chemical potential are in unhybridized *π * orbitals [9]. A key difference is that the electronic charge is not completely screened by the *sp*_2_ hybridization, shifting *π * orbitals by Δ _***n ***_= + Δ on *N * sites, and −Δ on *B * sites. This shift is the dominant cause of a gap of order 2Δ . Tight-binding fits to results from *ab-initio * simulations of monolayer BN have established the hopping *t *=2.33 eV [10], with an estimate of Δ =1.96 eV=0.84 *t *. The experiments indicate larger gaps: bulk h-BN has 5.971 eV [11], and monolayer h-BN has a gap of 5.56 eV [12] corresponding to Δ =2.78 eV=1.20 *t *. There is significant variation in phonon energies, $\hbar \Omega_{q}$, in h-BN [13]. Longitudinal acoustic (LA) phonon energies range up to around 140 meV at the *M * point, and transverse acoustic (TA) modes to around 110 meV at the *K * point. Optical phonon energies range between 160 and 200 meV. Coupling, *f *_***n ***_(***m ***), between electrons and phonons in either a polarisable substrate or the BN monolayer, is possible and the corresponding Hamiltonian is as follows:

(1) $$\begin{array}{lll} H & =-t\underset{\langle n,n^{\prime }\rangle \sigma }{\sum }(a_{n\sigma }^{†}c_{n^{\prime }\sigma }+c_{n^{\prime }\sigma }^{†}a_{n\sigma })-\underset{nm\sigma }{\sum }f_{n}(m)n_{n\sigma }\xi_{m}\; & \;\\ & \;+\underset{m}{\sum }\hbar \Omega (N_{m}+1/2)+\underset{n\sigma }{\sum }\Delta_{n}n_{n\sigma }.\; \end{array}$$

**Figure 1:**
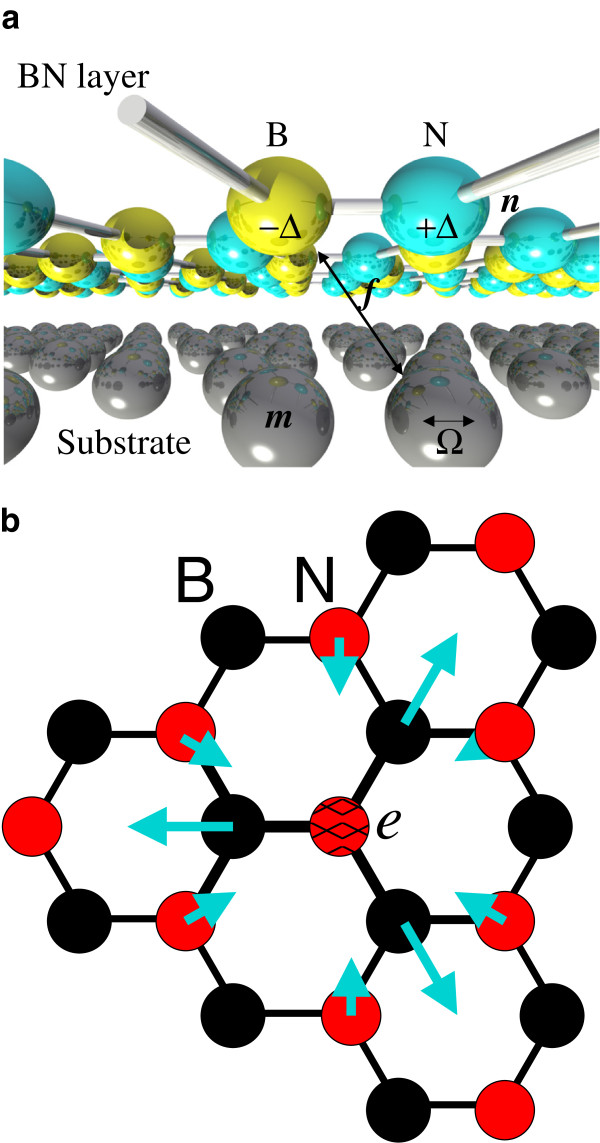
**Bn substrate system and interactions in a monolayer of BN.(a)** BN substrate system annotated with interactions. Electron-phonon interactions between the BN layer and substrate are poorly screened, and large interactions of strength *f *_***n ***_(***m ***) are possible. Ions in the substrate oscillate with frequency Ω. N sites have energy + Δ and B sites −Δ , opening a gap. The attractive phonon-mediated electronic interaction ***f *** binds electrons onto the same site, effectively enhancing the gap. **(b)** Interactions in a monolayer of BN. Red circles represent N atoms and black circles B atoms. Light blue arrows represent distortions expected from an excess of charge on the site labelled *e *.

The Hamiltonian terms are shown schematically in Figure 1 (a). $a_{n\sigma }^{†}$ creates electrons of spin *σ * on B sites and $c_{n^{\prime }\sigma }^{†}$ on N sites. Vectors ***n *** are to atoms in the monolayer, and ***m *** to atoms in the substrate. *N *_***m ***_ and *n *_***n ****σ *_ are the number operators for phonons and electrons respectively. *ξ *_***m ***_is the atomic displacement. The Hamiltonian is also approximately valid for interactions in the plane, and Figure 1 (b) shows the forces on ions from an increase in electron density at a B site. The largest forces are on the near-neighbor sites, so that the effective interaction is mainly site diagonal (electrons on A sites self-interact through phonons on B sites and vice versa). The diagram indicates that the strongest interaction is between electrons and optical phonon modes.

For simplicity, the Holstein electron-phonon interaction was used, $f_{n}(m)\propto \delta_{n,n^{\prime }}$, which qualitatively captures the physics. There may be quantitative changes to the results for longer range Fröhlich interactions and from modulation of the electron-phonon interaction due to incommensurability of the substrate, which was estimated at around ±8*% *of the average value [8].

## Results and discussion

The low order perturbation theory is applicable for low phonon frequency and weak coupling. A set of gap equations was derived by symmetrizing the self energy,

$$\Sigma (i\omega_{n})\approx \left(\begin{array}{cc} \mathrm{i\hbar}\omega_{n}(1-Z_{n}^{A})+{\bar{\Delta }}_{n}^{A} & 0\\ 0 & \mathrm{i\hbar}\omega_{n}(1-Z_{n}^{B})-{\bar{\Delta }}_{n}^{B} \end{array}\right).$$

The local approximation used here is a good starting point because the modulated potential Δ is large, and electrons are well localized. Off-diagonal terms do not feature in the lowest order perturbation theory for the Holstein model since the interaction is site diagonal. *Z *_*n *_ is the quasi-particle weight and ${\bar{\Delta }}_{n}$ is the gap function. For bosonic quantities, $\hbar \omega_{s}=2\pi k_{B}\mathrm{Ts}$ and for fermions, $\hbar \omega_{n}=2\pi k_{B}T(n+1/2)$. *T * is the temperature and *n * and *s * are integers.

The full Green function can be established using Dyson’s equation $G^{-1}(k,i\omega_{n})=G_{0}^{-1}(k,i\omega_{n})-\Sigma (i\omega_{n})$, leading to,

(2) $$G^{-1}(k,i\omega_{n})\;=\;\left(\;\begin{array}{cc} Z_{n}^{A}\mathrm{i\hbar}\omega_{n}-\Delta -{\bar{\Delta }}_{n}^{A} & \phi_{k}^{*}\\ \phi_{k} & Z_{n}^{B}\mathrm{i\hbar}\omega_{n}+\Delta +{\bar{\Delta }}_{n}^{B} \end{array}\;\right).$$

Substituting the expression for the Green function into the lowest order contribution to the self energy,

(3) $$\begin{array}{lll} \Sigma_{\mathrm{ii}}(k,i\omega_{n}) & =-k_{B}\mathrm{Tt}\lambda_{i}\underset{s}{\sum }\int \frac{{\text{d}}^{2}q}{V_{\mathrm{BZ}}}G_{\mathrm{ii}}\left(k-q,i\omega_{n-s}\right)\; & \;\\ & \;\times \left(d_{0}^{\left(\mathrm{ii}\right)}(q,\omega_{s})-2d_{0}^{\left(\mathrm{ii}\right)}(0,0)\right).\; \end{array}$$

Here, the phonon propagator, $d_{0}^{\left(\mathrm{ij}\right)}(q,\omega_{s})=\delta_{\mathrm{ij}}\Omega^{2}/(\Omega^{2}+\omega_{s}^{2})$; hence, there are no off-diagonal elements of the lowest order self energy. The use of a single averaged Ω and *λ *here is consistent with the mean-field approximation. At half-filling, it is reasonable to assume that *λ *_*A *_= *λ *_*B *_ so that Δ ^*A *^= Δ ^*B *^ and *Z *^*A *^= *Z *^*B *^. This leads to the gap equations,

(4) $${\bar{\Delta }}_{n}=-\mathrm{t\lambda}k_{B}T\underset{s}{\sum }\int \text{d}\varepsilon \frac{D(\varepsilon )\;{\Delta^{\prime }}_{n-s}(d_{0}(i\omega_{s})-2)}{\hbar^{2}\omega_{n-s}^{2}Z_{n-s}^{2}+{\Delta^{\prime }}_{n-s}^{2}+\varepsilon^{2}},$$

(5) $$Z_{n}=1-\frac{\mathrm{t\lambda}k_{B}T}{\omega_{n}}\sum_{s}\int \text{d}\varepsilon \frac{D(\varepsilon )\;\omega_{n-s}Z_{n-s}d_{0}(i\omega_{s})}{\hbar^{2}\omega_{n-s}^{2}Z_{n-s}^{2}+{\Delta^{\prime }}_{n-s}^{2}+\varepsilon^{2}},$$

where the full gap is ${\Delta^{\prime }}_{n}={\bar{\Delta }}_{n}+\Delta$. The density of states for a tight binding hexagonal lattice in the absence of a gap, *D *(*ε *), has the form given in reference [14]. The equations may be solved self-consistently by performing a truncated sum on Matsubara frequencies.

**Figure 2:**
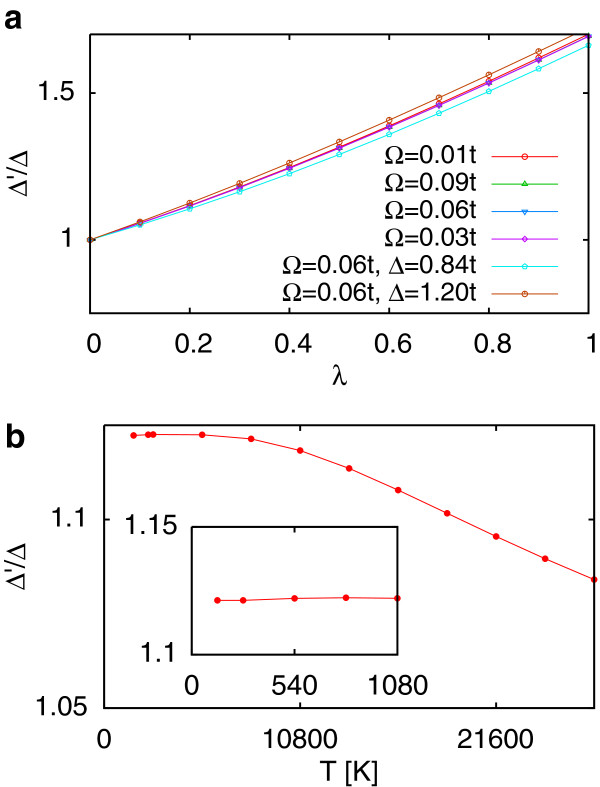
**Modification of the BN bandgap.(a)** The gap enhancement depends mainly on *λ *, is weakly dependent on Δ and shows almost no change with Ω. Calculations are made for Δ =*t *corresponding to a BN gap of 2Δ =4.66 eV, Δ =1.20*t *(2Δ =5.6 eV), and Δ =0.84*t *(2Δ =3.92 eV, the tight binding fit from reference [10]). *t *=2.33 eV, ℏΩ=0.01t=23 meV, ℏΩ=0.03t=70 meV, ℏΩ=0.06t=140 meV, ℏΩ=0.09t=210 meV, covering the full range of phonon frequencies in reference [13]. *k *_*B *_*T *=0.01*t *(*T *=268K) and *λ *≤1. **(b)** Variation of the gap with temperature, ℏΩ=0.05t=117 meV and *λ *=0.2. There is a weak temperature dependence due to the large Δ, consistent with the measurements in reference [15], with gap starting to close only for extremely high temperatures *T *>8,000 *K *, presumably above the melting point of the material. Red circles show the size of the gap, red lines are a guide to the eye.

Gap and quasi-particle weight functions only have a weak Matsubara frequency dependence (<0.3*% * for $\lambda =1,\;k_{B}T=\hbar \Omega =0.01t$). The local gap enhancement factors Δ ^*′ *^/Δ, are shown in Figure 2 (a) for various *λ *, showing a modest increase of around 70% for *λ *=1. The enhancement factor increases slightly with decreasing Δ but is essentially unchanged by modifications to phonon frequency and temperature for the parameter values used here. The temperature dependence of the gap was also calculated in Figure 2 (b). For very large temperatures, where *k*_B_*T* approaches Δ , there is a drop in the gap size. Below approximately 8,000 K (0.3*t *), this levels off, and the gap becomes relatively constant.

## Conclusions

A theory for the modification of BN band-gaps by interaction with phonons was presented here. It is of interest to make a comparison between the bandgaps of bulk h-BN, nanotubes, monolayer h-BN, and the theory presented here. Measured bandgaps of bulk h-BN are of between 5.8 eV [15] and 5.971 eV [11], indicating that interaction between layers increases the bandgap, consistent with the theory here. The bulk gap is also higher than that for nanotubes (5 eV) [16]. On the other hand, Song et al. [12] claim that the gap is reduced as BN thickness increases. The above discussion is presented with a warning that the theory requires that hopping between the substrate and the BN monolayer is small. Interlayer hopping will affect the bandwidth and bandgap, and the direct Coulomb interaction with strongly ionic substrates could also affect the band structure if the charge density at the surface of the substrate varies dramatically.

It is also of interest to estimate the magnitude of the bandgap modification due to electron-phonon interaction in isolated monolayers of BN. *Ab initio * calculations have attempted to quantify the magnitude of the interaction between electrons and acoustic phonons for small momentum excitations [17]. Extrapolating the interaction and taking a mean-field average (assuming mean momentum magnitude of 4*π */9*a *), the electron-phonon coupling can be estimated as $\lambda ={\left(4\pi /9\right)}^{2}E_{1}^{2}/2a^{2}t\bar{M}\Omega^{2}$, taking *E *_1_=3.66 eV from reference [17], $\bar{M}\approx 12.5$ amu, a=2.5Å. The mean energy of longitudinal acoustic phonons lies in the range of 50 to 75 meV, giving a range of *λ *=0.05 to 0.12, so the contribution of phonons to the bandgap is estimated as 3% to 7%. I would expect BN to have stronger interaction with optical phonons, since the pattern of distortions around an electronic defect is consistent with optical modes (see Figure 1).

The BN gap is too wide for digital applications. Recently, it has become possible to manufacture silicene, an atomically thick layer of silicon with similar properties to graphene [18], so it may be possible to make GaAs or AlP analogues to BN. Smaller gaps could be available from those materials, which might be used to create tunable bandgaps for atomically thick transistors.

## Competing interest

The author declares that he has no competing interest.

## Author’s information

JPH is a lecturer from the Faculty of Science, Department of Physical Sciences, The Open University, Walton Hall, Milton Keynes, UK.
